# Light conditions during Atlantic salmon embryogenesis affect key neuropeptides in the melanocortin system during transition from endogenous to exogenous feeding

**DOI:** 10.3389/fnbeh.2023.1162494

**Published:** 2023-04-21

**Authors:** Sissel Norland, Ana S. Gomes, Ivar Rønnestad, Jon Vidar Helvik, Mariann Eilertsen

**Affiliations:** Department of Biological Sciences, University of Bergen, Bergen, Norway

**Keywords:** *agrp1*, appetite, Atlantic salmon, *cart*, *npy*, photoperiod, *pomc*, yolk

## Abstract

During the first feeding period, fish will adapt to exogenous feeding as their endogenous source of nutrients is depleted. This requires the development of a functional physiological system to control active search for food, appetite, and food intake. The Atlantic salmon (*Salmo salar*) melanocortin system, a key player in appetite control, includes neuronal circuits expressing neuropeptide y (*npya*), agouti-related peptide (*agrp1*), cocaine- and amphetamine-regulated transcript (*cart*), and proopiomelanocortin (*pomca*). Little is known about the ontogeny and function of the melanocortin system during early developmental stages. Atlantic salmon [0–730 day degrees (dd)] were reared under three different light conditions (DD, continuous darkness; LD, 14:10 Light: Dark; LL, continuous light) before the light was switched to LD and the fish fed twice a day. We examined the effects of different light conditions (DD_*LD*_, LD_*LD*_, and LL_*LD*_) on salmon growth, yolk utilization, and periprandial responses of the neuropeptides *npya1, npya2, agrp1, cart2a, cart2b, cart4, pomca1*, and *pomca2*. Fish were collected 1 week (alevins, 830 dd, still containing yolk sac) and 3 weeks (fry, 991 dd, yolk sac fully consumed) into the first feeding period and sampled before (−1 h) and after (0.5, 1.5, 3, and 6 h) the first meal of the day. Atlantic salmon reared under DD_*LD*_, LD_*LD*_, and LL_*LD*_ had similar standard lengths and myotome heights at the onset of first feeding. However, salmon kept under a constant light condition during endogenous feeding (DD_*LD*_ and LL_*LD*_) had less yolk at first feeding. At 830 dd none of the neuropeptides analyzed displayed a periprandial response. But 2 weeks later, and with no yolk remaining, significant periprandial changes were observed for *npya1, pomca1*, and *pomca2*, but only in the LD_*LD*_ fish. This suggests that these key neuropeptides serve an important role in controlling feeding once Atlantic salmon need to rely entirely on active search and ingestion of exogenous food. Moreover, light conditions during early development did not affect the size of salmon at first feeding but did affect the mRNA levels of *npya1, pomca1*, and *pomca2* in the brain indicating that mimicking natural light conditions (LD_*LD*_) better stimulates appetite control.

## 1. Introduction

Atlantic salmon (*Salmo salar*) is the most successful aquaculture species in Norway and has a considerable economic impact (Iversen et al., 2020). In nature, the life-history transition of Atlantic salmon is closely linked to photoperiod with spawning in rivers taking place in the autumn, hatching in the spring, and the migratory smolt phase occurring in the late spring of the following years (Metcalfe and Thorpe, 1990). In aquaculture production, Atlantic salmon are typically reared in indoor freshwater facilities until they reach the end of smoltification when they are transferred to open net pens in seawater for the outgrowth phase. Under intensive rearing conditions, artificial light, and temperature are actively used to extend the day length and optimize production in indoor facilities compared to the natural environment (Villarreal et al., 1988; Stefansson et al., 1990; Berg et al., 1992; Handeland and Stefansson, 2001; Fjelldal et al., 2011).

Light is an important cue for the regulation of behavior, physiological functions, and rhythms in most vertebrates, including teleosts. In addition to retinal photoreception, which allows fish to visualize their surroundings, a high abundance of extraretinal photoreceptors is found in the central nervous system (Peirson et al., 2009; Perez et al., 2019). This is in line with the activation of specific brain regions following dark-to-light stimulation, such as the habenula, suprachiasmatic nucleus, thalamus, and hypothalamus in Atlantic salmon (Eilertsen et al., 2021). In the pineal organ, light affects melatonin production involved in the regulation of the circadian rhythm (Philp et al., 2000; Perez et al., 2019). The light-sensitive hypothalamus is also a key region for neuroendocrine systems such as reproduction and appetite (Philp et al., 2000; Sandbakken et al., 2012; Delgado et al., 2017; Perez et al., 2019). Indeed, several studies have shown that diurnal fish species have better growth under constant light (LL) (Villarreal et al., 1988; Stefansson et al., 1990; Berg et al., 1992; Jonassen et al., 2000; Ginés et al., 2004; Biswas et al., 2005; Fjelldal et al., 2011; Villamizar et al., 2013; Hou et al., 2019), likely because of the synergetic effect between feed intake and light. The photoperiod is considered the most important synchronizer of biological rhythms; but periodic feeding can also act as entrainment (Boulos and Terman, 1980; Mistlberger, 1994; Bolliet et al., 2001; Sanchez-Vazquez et al., 2019; Steindal and Whitmore, 2019).

Feed intake is also controlled by internal signals, in which several hormones and nerve signals are integrated and controlled centrally in the brain and affect the overall energy homeostasis and growth. When a salmon embryo and alevin feed on the yolk, it has an energetically closed system in which the nutrients in the yolk fully support the development, growth, and metabolism of the organism. The utilization rate of the yolk is limited by hydrolytic enzyme activities and the surface area of the syncytium layer (Skjærven et al., 2003; Kamler, 2008). To secure a steady supply of nutrients, salmon alevins initiate exogenous feeding while there is still some yolk matter left. The onset of exogenous feeding and regulation of appetite are critical for survival and are influenced by factors such as motivation, and prey type, as well as morphological features and integrated physiological functions for the detection, capture, ingestion, digestion, and assimilation of food particles (Rønnestad et al., 2013). During the first-feeding period, little is known about the dynamics of the appetite-controlling network or how the presence of endogenous yolk might influence it. Ontogenetic gene expression of some key peptides involved in appetite control has been investigated in some fish species, including Atlantic salmon (Moen et al., 2010), European eel (*Anguilla anguilla*) (Politis et al., 2018), giant grouper (*Epinephelus lanceolatus*) (Anderson et al., 2018), Atlantic cod (*Gadus morhua*) (Le et al., 2016), and Atlantic halibut (*Hippoglossus hippoglossus*) (Gomes et al., 2015, 2022). In first-feeding marine teleosts, several observations have shown that larvae continue to ingest food even though the gut is already full (Harboe et al., 2009; Rønnestad et al., 2013). This indicates that their satiety (anorexigenic) systems may not be fully developed in the first-feeding stages.

The melanocortin system is among the most studied and best-characterized networks for appetite control in vertebrates. This system is characterized by two major hypothalamic neuronal circuits known to stimulate (orexigenic) or inhibit (anorexigenic) appetite (Schwartz et al., 2000). Key neuropeptides of the melanocortin pathway include neuropeptide y (*npy*), agouti-related peptide (*agrp1*), cocaine- and amphetamine-regulated transcript (*cart*), and proopiomelanocortin (*pomc*) that are shown to respond to fed/fasted state in salmon (Murashita et al., 2009a,^2011^; Valen et al., 2011; Kalananthan et al., 2020b,^2021^, ^2023^; Tolås et al., 2021). Because of whole-genome duplication events, Atlantic salmon possess multiple paralogs for most genes (Allendorf and Thorgaard, 1984; Lien et al., 2016). The topological distribution of *npya*, *agrp1*, *cart2b*, and *pomca* has recently been mapped in Atlantic salmon demonstrating that these neuropeptides are expressed in the tuberal hypothalamus, the putative homolog of the mammalian arcuate nucleus, indicating that this is a key region for appetite regulation in teleosts (Cerdá-Reverter and Peter, 2003; Cerdá-Reverter et al., 2003; Norland et al., 2023).

How, and to what extent different light conditions during the endogenous phase affect the exogenous feeding period is not fully understood. Light and feeding regimes may influence the internal clock, which may in turn impact the appetite dynamics. Therefore, eggs and alevins kept under constant conditions lack zeitgeber, in contrast to individuals kept under a light-dark periodicity that mimics natural light conditions. How this affects the regulatory function of the melanocortin system in first-feeding alevins is not known. This study aimed to describe the effects of three different light conditions (LL, LD, and DD) during development on the mRNA expression of neuropeptides *npya1, npya2, agrp1, cart2a, cart2b, cart4, pomca1*, and *pomca2* before and after a meal during the first feeding period in Atlantic salmon, as well as its effects on the fish growth. Based on the results we propose that the activation of key neuropeptides in the brain is linked to the depletion of the yolk content.

## 2. Materials and methods

### 2.1. Ethical statement

All animal treatments were performed according to the national animal welfare legislation and complied with the ARRIVE guidelines (Percie du Sert et al., 2020). As fish did not undergo handling except euthanasia, no special approval was required according to Norwegian National legislation via the Norwegian Animal Welfare Act (LOV-2015-06-09-16-65) and Regulations on the Use of Animals in Experiments (FOR-2017-04-05-451), given by the EU (Directive 2010/63/EU) for animal experiments. All fish were euthanized with metacaine (MS-222™; MSD Animal Health, Netherlands) on-site, before further handling. The trials were conducted at an approved laboratory facility by the Norwegian Food Safety Authority (VSID 2135) at Bergen High Technology Center (University of Bergen, Bergen, Norway). All personnel involved in the experiment had undergone training (FELASA-C) approved by the Norwegian Food Safety Authority, which is mandatory for running experiments involving animals included in the Animal Welfare Act.

### 2.2. Experimental design

**FIGURE 1:**
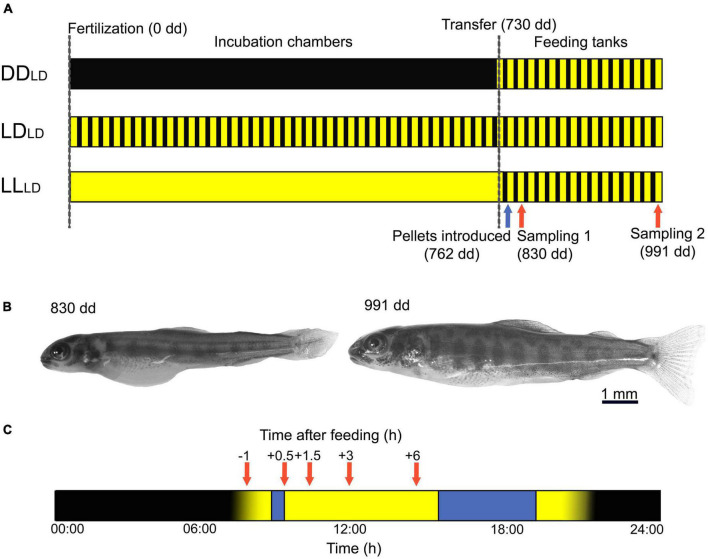
Experimental design and sampling of first feeding Atlantic salmon. **(A)** Atlantic salmon were reared under three different light conditions: constant darkness (DD), 14 h light-10 h dark (LD), and constant light (LL) from fertilization (dashed line, 0 dd) until 1 week before the onset of exogenous feeding when alevins were transferred to feeding tanks (dashed line, 730 dd). In the feeding tanks, the light was set to 14:10 LD for all tanks, and pellets were first introduced at 762 dd (blue arrow). Fish were sampled at 1 (830 dd) and 3 weeks (991 dd) after the onset of exogenous feeding (red arrows). **(B)** First-feeding Atlantic salmon alevin with yolk sac at 830 dd; and Atlantic salmon fry with clear cryptic (spotted) coloration at 991 dd. **(C)** The timeline of the sampling days is shown in hours, from 00:00 to 24:00. The light was switched on at 08:00. Fish were fed twice a day (blue box) and sampled (red arrows) before (−1 h) and after (0.5, 1.5, 3, and 6 h) the first meal of the day (9:00–9:30). Blue box: Feeding period. Red arrows: sampling time points. Yellow: Lights on. Black: Lights off. dd, day-degrees.

Two sibling groups of Atlantic salmon eggs and sperm were obtained from Mowi (Tveitevågen, Askøy, Norway) and transferred to a wet lab at the Bergen High Technology Center where eggs were fertilized. The fertilized eggs were randomly divided into three light conditions: continuous darkness (DD), light-dark photoperiod (14:10 LD, 0.1 W/m^2^), and continuous light (LL, 0.1 W/m^2^) and incubated in racks in egg incubator chambers (45 × 45 × 45 cm, 500 eggs/racks) at 5.7 ± 0.5°C (2 replicate chambers × 3 light conditions, 6 tanks in total; Figure 1A). Salmon development was estimated based on the number of day-degrees (dd) from fertilization. One week before the start of exogenous feeding (730 dd), the fish in each light treatment were transferred into feeding tanks (Ø = 60 cm, 3 replicates × 3 tanks, 9 tanks in total). In the feeding tanks, the light was set at 14:10 LD for all tanks at an intensity of 1.0 W/m^2^ until the end of the experiment. The temperature was increased from 5.7 ± 0.5°C to 11.5 ± 0.3°C at 730 dd. Fish were fed from 762 dd with a commercial diet (EWOS Micro starter diet, 0.6 mm) twice a day (09:00–09:30 and 16:00–20:00). The amount of feed per day was 4% of the total biomass per tank (total fish wet weight) + 25% excess feed. Of this, 25% was given in the morning and 75% in the afternoon. At 21:30 the day before sampling, the excess feed from the afternoon meal was manually removed from the tanks. The DD_*LD*_, LD_*LD*_, and LL_*LD*_ refer to the light treatment applied during the endogenous, and subscript letters refer to the exogenous feeding stages.

### 2.3. Sampling

To evaluate the effect of light on the fish growth, yolk utilization, and mRNA profile of appetite-controlling genes, fish samples were collected 1 and 3 weeks after the onset of exogenous feeding—830 dd (5 days after exogenous feeding started and some yolk sac remaining) and 991 dd (19 days after feed were introduced and fish yolk sac fully consumed) (Figure 1B). Three fish per tank (3 fish × 9 tanks) per sampling point [1 h before feeding (BF) and 0.5, 1.5, 3, and 6 h after feeding (AF) the first meal of the day (Figure 1C)] were collected and sacrificed with a lethal dose of MS-222 (200 mg/L). The fish were photographed (NIKON D7500) to measure standard length (SL), myotome height (MH), and yolk sac height, length, and surface area. The head was cut and transferred to RNAlater (Invitrogen, Carlsbad, CA, USA), kept at 4°C overnight, and stored at −80°C. In total, 270 fish were collected in the two sampling days.

### 2.4. Growth and yolk utilization

Assessment of standard length (SL), myotome height (MH), and yolk sac height, length, and surface area were performed using ImageJ (NIH, Bethesda, MD, USA,^1^ RRID:SCR_003070). The specific growth rate (SGR) was calculated using the average SL and MH for each light condition group using Equation 1, where *g1* is the average value at 830 dd and *g2 is* the average value at 991 dd and *Δt* is the elapsed days between 830 and 991 dd (14 days) (Crane et al., 2020). Yolk length (Ysl) and height (Ysh) were measured to estimate the yolk sac volume using Equation 2 (Rønnestad et al., 1992).


Equation⁢ 1:S⁢G⁢R=100×((g⁢2g⁢1)1△⁢t-1)

Equation⁢ 2:Y⁢o⁢l⁢k⁢s⁢a⁢c⁢v⁢o⁢l⁢u⁢m⁢e=π6×Y⁢s⁢l×Y⁢s⁢h2

### 2.5. RNA isolation and cDNA synthesis

Atlantic salmon heads were thawed on ice, and the brains were dissected out of the skull and transferred into new RNAlater. Total RNA was extracted using TRI-reagent (SigmaMillipore, MO, USA) according to the manufacturer’s protocol. RNA quantity, purity, and integrity were analyzed using a Nanodrop ND-1000 spectrophotometer (Thermo Fisher Scientific, Waltham, MA, USA; RRID:SCR_016517) and a 2100 Bioanalyzer (Agilent Technologies, CA, USA; RRID:SCR_018043) with a RNA 6000 Nano Kit (Agilent). DNase treatment and cDNA synthesis were carried out using Quantitect Reverse Transcription kit (cat. # 205313, Qiagen, Germany) according to the manufacturer’s protocol.

### 2.6. Quantitative RT-PCR

The mRNA expression of *npya1, npya2, agrp1, cart2a, cart2b, cart4, pomca1*, and *pomca2* were analyzed in Atlantic salmon whole brains. The genes in this study were chosen based on their importance in appetite control and response to feeding in Atlantic salmon parr and post-smolt (Murashita et al., 2009a,^2011^; Valen et al., 2011; Kalananthan et al., 2020a,b, 2021, 2023; Tolås et al., 2021) and their topological distribution in the parr brain (Norland et al., 2023). Primers for the selected neuropeptides were based on previous studies in Atlantic salmon (Kalananthan et al., 2020a,^2021^; Tolås et al., 2021; Supplementary Table 1). The primer pairs efficiency was determined using a standard curve dilution series (10-fold) generated using each target gene cloned into the pCR4-TOPO vector (Thermo Fisher Scientific). Each assay had an efficiency between 95–103% and R^2^ between 0.996–0.999. qPCR assays were performed using 10 μl of iTaq Universal SYBR Green Supermix (Bio-Rad, Hercules, CA, USA), 0.4 μM of each forward and reverse primer, 8 μl cDNA template (between 12.5–50 ng), and Ultrapure water (Biochrom, Berlin, Germany) to a total volume of 20 μl reaction. Assays were run on a Bio-Rad CFX96™ Real-Time System (RRID:SCR_018064) under the following cycling conditions: 95°C for 30 s; 40 cycles of 95°C for 5 s, and 60°C for 25 s. Melting curve analysis was performed over a range of 65–95°C (increment of 0.5°C for 2 s). Three controls were included in each plate (no template, minus reverse transcription control and between-plate control). The copy number for each gene was calculated based on the specific standard curve using Equation 3. The copy number was normalized using the ng of total RNA used for the assay (copy number/ng of total RNA) and further normalized to the copy number of the reference gene ribosomal protein s20 (*rps20*).


Equation⁢ 3:c⁢o⁢p⁢y⁢n⁢u⁢m⁢b⁢e⁢r= 10C⁢q-i⁢n⁢t⁢e⁢r⁢c⁢e⁢p⁢ts⁢l⁢o⁢p⁢e

### 2.7. Statistical analysis

Data exploration was conducted to identify outliers and evaluate normality. A generalized linear mixed model (GLMM) with gamma distribution (log-link function) was used to model SL and MH as a function of light *L* and age *A* (Equation 4), the yolk sac surface area and yolk sac volume as a function of *L* at age 830 dd [no yolk was left at age 991 dd (Equation 5)], and the target gene relative expression as a function of light *L*, feeding *F* and age *A* (Equation 6). Tanks were added as random intercepts to account for possible tank effects.


Equation⁢ 4:n⁢(Bi,L,A,t)=b0,t+β1⁢Li,t+β2⁢Ai,t+εi

Equatio⁢n⁢ 5:n⁢(Yi,L,t)=b0,t+β1⁢Li,t+εi

With *B* as the biometry parameters SL or MH in fish *i* at age *A* 830 dd or 991 dd and light condition *L*, and *Y* as the yolk sac area or volume at age 830 dd; *b*_0,*t*_∼*N*(0,σ_*I*_) as random intercepts for tank *t* to represent potential differences between tanks (Supplementary Figure 1); β_1_*L*_*i*,*t*_ as fixed effect of light (factor) and β_2_*A*_*i*_ as fixed effect of fish age (factor).


Equation⁢ 6:n⁢(Ei,L,F,A,t)=b0,t+β1⁢Ai,t+β2⁢Li,t+β3⁢Fi,t

$$+\beta_{4,F_{i,t},}\,L_{i,t}+\beta_{5,F_{i,t}}\,A_{i,t}+\varepsilon_{i}$$

With *E* as the target gene expression by fish *i* at age *A* 830 dd or 991 dd and light condition *L*; *b*_0,*t*_∼*N*(0,σ_*I*_) as random intercepts for tank *t* to represent potential differences between tanks (Supplementary Figure 2). In addition to β_1_*A*_*i*,*t*_ as fixed effect of age (factor), β_2_*L*_*i*,*t*_ as a fixed effect of light (factor), and β_3_*F*_*i*,*t*_ as fixed effect of feeding time (factor), interaction terms between feeding time and light β_4,*F*_*i*,*t*_,_*L*_*i*,*t*_ and between feeding time and age β_5,*F*_*i*,*t*_,_*A*_*i*,*t*_ were also included. The interactions were added to account for the potential dependency of the effect of light and feeding and between age and feeding. The error distribution was assumed to be Gamma-distributed ε_*i*_*Gamma*(λ) with log-link.

Backward selection was applied based on the Akaike information criterion. The models were evaluated by the residual distributions. *Post-hoc* tests with Tukey approximation were used to analyze pairwise differences and contrasts between groups (light, feeding, and age). Statistical significance was set at *p* < 0.05. All data are presented as mean ± 95% confidence interval. Data exploration and statistical analyses were performed in RStudio (RRID:SCR_000432)^2^ R.4.1.1 with the packages *glmmTMB* (Brooks et al., 2017) to fit the model in Equations 4, 5 and 6, *DHARMa* (Hartig, 2022) was used to validate the model fit, (simulated) residual distribution and to evaluate residual distributions, and *emmeans* (Lenth et al., 2018) for *post-hoc* testing.

## 3. Results

### 3.1. Effect of light during development on growth

**FIGURE 2:**
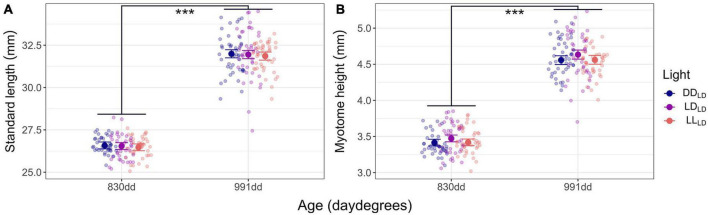
Model predicted values and raw data of growth in first feeding Atlantic salmon at 830 dd and 991 dd. **(A)** Standard length (mm). **(B)** Myotome height (mm). The larger dots and lines indicate model-predicted means and 95% confidence intervals, respectively, for each age. Transparent dots represent individual raw data points. Per light condition group at 830 dd *n* = 41 for DD_LD_ and LD_LD_, and *n* = 42 for LL_LD_. Per light condition group at 991 dd *n* = 44 for DD_LD_ and LL_LD_, and *n* = 42 for LD_LD_. Asterisks show the significant level (*** indicates *p* < 0.001).

The Atlantic salmon grew significantly (*p* < 0.0001) in SL and MH between the first (830 dd) and second sampling (991 dd) for all light conditions (Figure 2; Table 1). No significant differences caused by light conditions during development were observed for SL or MH in first-feeding salmon at 830 dd or 991 dd. There were no observable differences for the mean specific growth rate (SGR) between 830 dd and 991 dd for SL or MH between light conditions (Table 2).

**TABLE 1:** Morphometric data for first feeding Atlantic salmon at 830- and 991-day degrees (dd) (1 and 3 weeks after offered feed for the first time).

Age	830dd			991dd		
**Light regime**	**DD_LD_**	**LD_LD_**	**LL_LD_**	**DD_LD_**	**LD_LD_**	**LL_LD_**
SL (mm)	26.6 ± 0.71	26.5 ± 0.71	26.5 ± 0.6	31.9 ± 1.15	32.0 ± 1.49	31.8 ± 0.84
MH (mm)	3.39 ± 0.14	3.48 ± 0.22	3.44 ± 0.18	4.59 ± 0.24	4.63 ± 0.33	4.53 ± 0.19
Ysa (mm^2^)	19.9 ± 2.68	20.7 ± 2.66	19.2 ± 2.65	–	–	–
Ysv (mm^3^)	41.3 ± 9.80	45.4 ± 9.11	40.6 ± 8.20	–	–	–

Larvae were reared under continuous light (LL), 14 h light-10 h darkness (LD), and continuous darkness (DD) until 730 dd, when all fish were transferred to an LD regime. The data are presented as mean ± sd. SL, standard length; MH, myotome height; Ysa, yolk sac surface area; Ysv, yolk sac volume.

**TABLE 2:** Morphometric changes in first-feeding Atlantic salmon from 830 to 991-day degrees (dd) (14 days in between samplings).

Light regime	DD_LD_	LD_LD_	LL_LD_
SL SGR (% day^–1^)	1.31	1.36	1.31
MH SGR (% day^–1^)	2.19	2.06	1.99

Larvae were reared under continuous light (LL), 14:10 L:D, and continuous darkness (DD) until 730 dd, when all fish were transferred to an LD (14:10) regime. SGR, specific growth rate; SL, standard length; MH, myotome height.

### 3.2. Effect of light on yolk utilization

**FIGURE 3:**
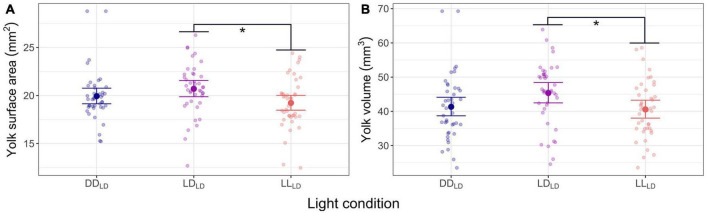
Model predicted values and raw data of yolk sac in first feeding Atlantic salmon at 830 dd for the three light conditions (DD_LD_, LD_LD_, and LL_LD_). **(A)** Yolk sac surface area (mm^2^). **(B)** Yolk sac volume (mm^3^). The larger dots and lines indicate model-predicted means and 95% confidence intervals, respectively, for each light condition group. Transparent dots represent individual raw data points. Per light condition groups *n* = 41, for DD_LD_ and LD_LD_, and *n* = 42 for LL_LD_. Asterisks show the significant level (* indicates *p* < 0.05).

One week into the first feeding (830 dd), the LD_*LD*_ group had a significantly larger yolk sac surface area (*p* = 0.034) and volume (*p* = 0.049) than the LL_*LD*_ fish group (Figure 3; Table 1). A similar trend was found between LD_*LD*_ and DD_*LD*_ albeit non-significant. The yolk sac was fully consumed between 1 and 3 weeks after starting exogenous feeding (830 dd and 991 dd) for all light condition groups.

### 3.3. Light effect on the mRNA expression of appetite-controlling neuropeptides

**FIGURE 4:**
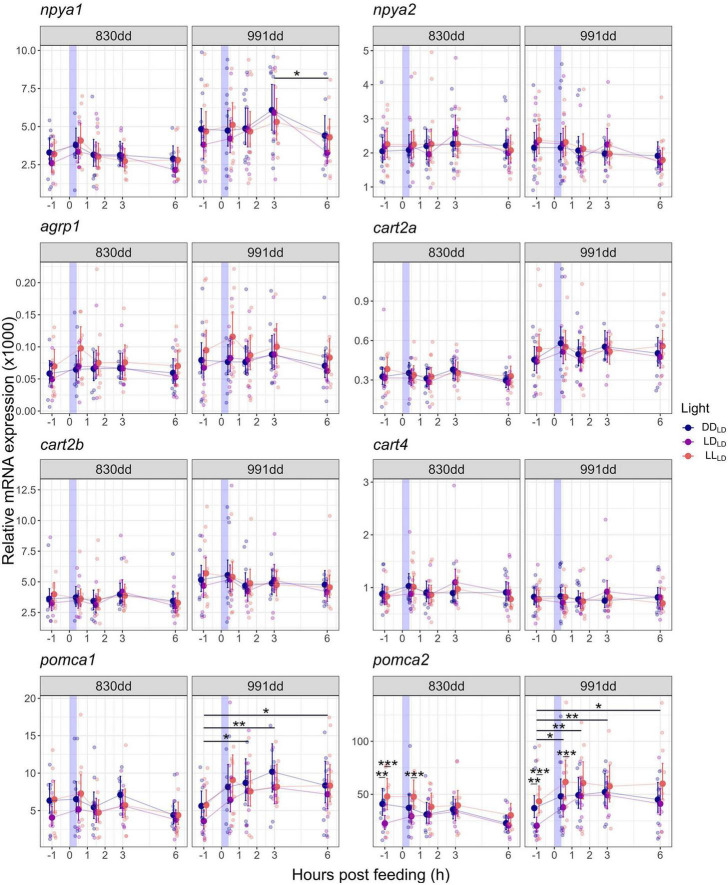
Model predicted values and raw data of relative mRNA expression of *npya1*, *npya2*, *agrp1*, *cart2a*, *cart2b*, *cart4*, *pomca1*, and *pomca2* in Atlantic salmon whole brain 1 week (alevins, 830 dd) and 3 weeks (fry, 991 dd) into exogenous feeding in response to a meal and light condition during development. Larger dots and lines indicate model-predicted means and 95% confidence intervals, respectively, for each sampling point and light condition. Smaller, transparent dots represent individual raw data points. Asterisks show the significant level (*p < 0.05, **p < 0.01, *** *p* < 0.001). Please refer to Supplementary Table 3 for the number of individuals.

All target genes were expressed in the brain 1 week into the first feeding period in Atlantic salmon when the alevins (830 dd) still had yolk sac (Figure 4; Supplementary Figure 3). Light affected the mRNA expression of *pomca2* in fish kept in LD_*LD*_, which had a significantly (*p* < 0.01) lower expression in unfed fish (1 h before a meal) compared to the LL_*LD*_ and DD_*LD*_ fish (Figure 4; Supplementary Table 2). At 0.5 h after feeding the mRNA expression of *pomca2* in fish kept in LD_*LD*_ was significantly lower compared to the LL_*LD*_ fish group (Figure 4; Supplementary Table 2). No significant differences were found between DD_*LD*_ and LL_*LD*_ groups in the *pomca2* mRNA expression at 830 or 991 dd (Figure 4). No significant changes were found for *npya1, npya2, agrp1, cart2a, cart2b, cart4*, or *pomca1* mRNA expression between the light conditions.

### 3.4. mRNA expression of appetite-controlling neuropeptides in response to a meal

The overall expression of central appetite-related neuropeptides in response to a meal was analyzed 1 h before feeding (BF), and 0.5, 1.5, 3, and 6 h after feeding (AF) in the brain of alevins and fry. For alevins (at 830 dd), no effect of feeding was observed on the mRNA expression of the target genes in any of the light groups (Figure 4; Supplementary Table 2). In the fry (991 dd), the relative expression of *npya1, pomca1*, and *pomca2* in response to a meal were significantly (*p* < 0.05) affected in the LD_*LD*_ group. In the LL_*LD*_ and DD_*LD*_ groups, the mRNA levels of these genes remained unchanged over time (BF and AF). For the LD_*LD*_ fry, *npya1* remained unchanged between 1 h BF until 3 h AF, before significantly dropping between 3 and 6 h AF (*p* = 0.015) (Figure 4; Supplementary Table 2). *pomca1* in LD_*LD*_ increased after feeding until 1.5 h AF (*p* = 0.014) and remained significantly higher until 6 h AF than the unfed fry (BF) (*p* < 0.05) (Figure 4; Supplementary Table 2). *pomca2* showed a similar response to *pomca1* in the LD_*LD*_ fry, increasing significantly (*p* < 0.05) in fed fry (0.5–6 h AF) compared to unfed (Figure 4; Supplementary Table 2). No significant changes were found for *npya2, agrp1, cart2a, cart2b*, and *cart4* mRNA expression.

## 4. Discussion

This study investigated the effects of three different light conditions during Atlantic salmon development on growth, yolk utilization, and mRNA expression levels of key neuropeptides in the melanocortin system during the transition from endogenous to exogenous feeding. Results showed differences in terms of yolk consumption and activation of the mRNA transcription of the neuropeptides in the brain between light conditions. Additionally, the periprandial mRNA expression for some of the neuropeptides differed between the two stages (830 dd versus 991 dd) and between light conditions.

### 4.1. Effect of light on growth

In this study, Atlantic salmon kept under different light conditions during the yolk sac utilization period and equal LD conditions during the first-feeding period (830 dd or 991 dd), showed no significant differences on growth, i.e., SL or MH (Figure 2; Table 1). Thus, light conditions before first feeding seem to not directly affect salmon growth performance during their early development. In contrast, if the light conditions used during development were continued through the exogenous feeding, growth differences would be expected. Several studies have shown that diurnal fish species, including Atlantic salmon, have stronger growth after the onset of exogenous feeding when reared under long days or continuous light (LL) (Villarreal et al., 1988; Stefansson et al., 1990; Berg et al., 1992). However, fast-growing fish kept under LL often display a higher proportion of malformations, such as overinflated swim bladders and cranial deformities (Villamizar et al., 2009, 2014), however, no deformities were observed in the fish sampled in our study. RNA sequencing of whole salmon embryos and alevins has shown that non-visual opsins, which are light-sensing proteins with non-visual functions, are present from an early stage (255 dd) (Eilertsen et al., 2022b). At older stages (parr) these non-visual opsins are expressed in the central nervous system including the pineal organ and deep brain (Philp et al., 2000; Peirson et al., 2009; Sandbakken et al., 2012; Perez et al., 2019). The early expression of non-visual opsins in salmon (Eilertsen et al., 2022b) supports that salmon eggs and alevins can receive different light cues during endogenous feeding; however, based on the results of this study, this does not influence alevin growth during the first feeding period.

Most fish are visual feeders around first feeding, which means that they detect and ingest food particles that they can see (Rønnestad et al., 2013; Nikolaou and Meyer, 2015; Eilertsen et al., 2022a). First-feeding Atlantic salmon use ram feeding during the first days of exogenous feeding but gradually change towards suction-feeding, which is a more effective method to catch food supporting their growth (Coughlin, 1991). Here, after 3 weeks of exogenous feeding, no differences in growth were observed between the light condition groups. Our results suggest that the first-feeding Atlantic salmon may maintain equal growth from alevins to fry independent of previous light conditions during development. This is in line with a previous study demonstrating that salmon growth can be leveled out by an increase in food consumption regardless of the light conditions they are exposed to during early life (Villarreal et al., 1988). This indicates the ability of salmon to allocate resources toward somatic growth when the fish is in a surplus of energy.

### 4.2. Utilization of yolk reserves

In this study, salmon reared in constant light conditions (DD_*LD*_ or LL_*LD*_) had a faster yolk sac utilization rate during endogenous feeding compared to LD_*LD*_, which mimics natural conditions. Fish larvae reared under long days and high light intensity seem to have greater metabolism and yolk sac utilization compared to fish kept on short days or low light intensity (Downing and Litvak, 2002; Finn et al., 2002; Finn and Rønnestad, 2003). For example, haddock (*Melanogrammus aeglefinus*) larvae reared under high-intensity light (18 μmol s^–1^ m^–2^) had significantly reduced yolk sac area, which was associated with increased larval activity during light, elevated embryonic metabolism, and growth resulting in increased consumption of endogenous energy reserves (Downing and Litvak, 2002). Moreover, Atlantic cod and turbot (*Scophthalmus maximus*) metabolism is significantly affected by light at an early age (Finn et al., 2002; Finn and Rønnestad, 2003). Our results showed that Atlantic salmon reared under DD_*LD*_ and LL_*LD*_ have enhanced energy requirements during endogenous feeding demonstrated by a smaller yolk sac at 830 dd. This is in line with a transcriptomic study on Atlantic salmon reared under different photoperiods (LD, DD, and LL) prior to exogenous feeding, which demonstrated an increased muscle activity and use of energy in constant light conditions compared to LD (Eilertsen et al., 2022b). As in Atlantic salmon, European sea bass (*Dicentrarchus labrax*) absorbed the yolk faster in LL, but slower in DD (Villamizar et al., 2009) contrasting to what was observed here for Atlantic salmon. Previous studies have found that photoperiod manipulation induces an enhanced stress response in fish (Leonardi and Klempau, 2003; Villamizar et al., 2014; Corona-Herrera et al., 2022), but not in all fish species (Biswas et al., 2004). However, how stress may have affected metabolism and yolk utilization in the present study could not be determined.

### 4.3. Effect of age and light on the mRNA expression of appetite-controlling neuropeptides

Despite the significant industrial importance of Atlantic salmon, little is known about when systems controlling appetite and energy homeostasis are fully established. This study demonstrated that key neuropeptides of the melanocortin system are expressed in the Atlantic salmon brain at first feeding. This is in line with a previous study showing that *npya1, agrp1, cart2b*, and *pomca1* are expressed in the head region of newly hatched Atlantic salmon [named *npy, agrp, cart*, and *pomca1* in Moen et al. (2010)]. The fact that the genes encoding key neuropeptides involved in appetite control are expressed before the first-feeding window is most likely linked with a hardwired activation of systems required for sufficient functionality during the first-feeding period. This also supports the hypothesis that these neuropeptides have roles besides appetite control, as demonstrated by *agpr1* which is required for normal growth in zebrafish (*Danio rerio*) larvae (Zhang et al., 2012).

During ontogeny, the expression level of neuropeptides usually increases as a result of an increased number of neurons, as demonstrated in the development of Atlantic salmon (Moen et al., 2010), Atlantic cod (Le et al., 2016), and zebrafish (Mukherjee et al., 2012). In the present study, *pomca2* was lower expressed in the LD_*LD*_, which approximates natural light conditions, compared to the continuous light conditions (DD_*LD*_, and LL_*LD*_) before a meal was given (Figure 4). This result may not be solely a response to appetite, given that *pomca2* is a precursor peptide that is cleaved into several peptides with varied functions (Takahashi and Mizusawa, 2013). Lower *pomca2* leads to reduced mRNA translated in peptide synthesis. As a result, the difference in *pomca2* 1 h BF may influence endogenous changes, such as plasma cortisol which overall influences the food intake. The differences in *pomca2* expression at 1 h BF might be a result of a light-dependent modulation of the “metabolic set-point” of the hypothalamus. The set-point is an important factor in the hypothalamic response to the adrenocorticotropic hormone, a peptide derived from *pomc* cleavage (Henry et al., 2010; Walton et al., 2011). Precisely how the metabolic set-point is established is not fully understood but might involve feedback from photoperiod, melatonin, leptin, and short-term satiation signals (Morgan and Mercer, 2001; Walton et al., 2011). Extraretinal photoreceptors affect endocrine rhythms, i.e., melatonin, glucocorticoids, and thyroid hormone cycles (Perez et al., 2019). Several studies have shown that thyroid hormones play a role in appetite regulation (Kong et al., 2004; Walton et al., 2011; Deal and Volkoff, 2020; Wasserman-Bartov et al., 2022). As a result, light may indirectly affect the metabolic set-point.

### 4.4. Light conditions during embryogenesis influence the mRNA expression of appetite-controlling neuropeptides in response to a meal

Several reports have suggested that *agrp1, cart2b*, and *pomca1* have anorexigenic roles in Atlantic salmon parr (Murashita et al., 2009a,^2011^; Valen et al., 2011). In contrast, *npya* seemed to serve an anorexigenic effect after a meal, but it also had an orexigenic role after 6 days of fasting (Murashita et al., 2009a; Valen et al., 2011). In Atlantic salmon post-smolt, fasting influences the hypothalamic mRNA levels of *agrp1, pomca2, cart2b, cart2a, npya1*, and *npya2* (Kalananthan et al., 2020b,^2021^, ^2023^; Tolås et al., 2021). Fasting significantly increased the hypothalamic *agrp1*, and the expression was negatively correlated with stomach filling, indicating an orexigenic role in post-smolts (Kalananthan et al., 2020b). In the same study, hypothalamic *pomca2* expression was positively correlated with stomach filling (though non-significant), indicating an anorexigenic role. Hypothalamic and midbrain *npya2*; and hypothalamic, midbrain, and olfactory bulb *cart2b* mRNA increased during 3–4 days of fasting indicating orexigenic effects by these neuropeptides (Kalananthan et al., 2021; Tolås et al., 2021). During long-term (4 weeks) fasting, hypothalamic *npya1, cart2a*, and *cart2b* displayed an orexigenic role (Kalananthan et al., 2023).

Different light conditions during endogenous feeding have affected the mRNA expression profiles of key appetite-controlling neuropeptides in response to a meal during the first-feeding period. However, periprandial alterations in *npya1, pomca1*, and *pomca2* mRNA levels were only found in 991 dd fish reared under LD_*LD*_, which emulates natural light conditions (Figure 4). The LD-regime is an environmental cue used to entrain endogenous rhythms and, thus, fish kept in LD exhibit greater rhythmic amplitudes (Isorna et al., 2017). In our study, only LD_LD_ had a significant periprandial response, suggesting that photoperiod and endogenous appetite may be synchronized during development. The expression of *npya1* significantly decreased between 3 h AF and 6 h AF, indicating either a post-prandial response to the morning meal, or a pre-prandial response to the second meal of the day. This result is in line with the increased whole-brain *npya* expression in the hours after feeding in Atlantic salmon parr maintained under 12:12 LD (Valen et al., 2011). However, whole-brain *npya* expression after 6 days of fasting in Atlantic salmon parr indicated an orexigenic role (albeit non-significant) (Murashita et al., 2009a). Spatial distribution analysis have demonstrated that *npya* mRNA is expressed in the lateral tuberal hypothalamus neighboring *agrp1*-positive cells, indicating an important hypothalamic region in appetite control in salmon (Norland et al., 2023). Hypothalamic *npya1* increased during long-term fasting indicating an orexigenic effect under a natural simulated photoperiod, while *npya2* displayed an orexigenic trend after 4 days of fasting, albeit non-significant, under continuous light (Tolås et al., 2021; Kalananthan et al., 2023). In salmon, *npya* is also expressed in several brain regions, including the telencephalon, optic tectum, and thalamus (Norland et al., 2023). Thus, *npya* is present in brain regions linked with sensory inputs driving behavioral modulations and food intake (Filosa et al., 2016; Chen et al., 2018; Wee et al., 2019, 2022; Corradi and Filosa, 2021; Norland et al., 2023). In this study, the *npya1* expression was higher than that of *npya2* during the first-feeding period, which is in line with older stages (Tolås et al., 2021). The duplication of *npya* is a result of the salmon id-specific genome duplication (Tolås et al., 2021). Thus, the periprandial responses of *npya1* and *a2* might be a stage-specific subfunctionalization, reflecting possible neural plasticity in Atlantic salmon allowing the fish to adapt to different environmental conditions during different life stages.

In the current study, *pomca1* and *a2* showed a clear anorexigenic effect in the LD_LD_ group, with significantly higher mRNA expression after feeding at 991 dd (Figure 4). The main sites of *pomca* expression are the adenohypophysis and basal hypothalamus (Norland et al., 2023). Increased whole-brain *pomca1* expression was observed after feeding in Atlantic salmon parr kept under 12:12 LD, but no change in *pomca2* expression was observed between fasted and fed states (Valen et al., 2011). Atlantic salmon post-smolt kept under a natural simulated photoperiod had elevated expression of hypothalamic *pomca1* and *a2* after 6 weeks of fasting, indicating an orexigenic effect (Kalananthan et al., 2023).

### 4.5. Behavioral aspects and transition to exogenous feeding

To be successful, alevins and fry need to detect, capture, ingest, digest, and assimilate food items for exogenous feeding (Rønnestad et al., 2013). The feeding state of fish modulates the activity of sensory processing involved in fine-tuning the response to external stimuli, such as prey capture or avoidance behavior (Corradi and Filosa, 2021). Thus, we speculate that the first-feeding salmon need a motivator (i.e., hunger or desire to eat) that will influence their behavior and locomotor performance during food capture. An example is that increased swimming activity will decrease the time to capture food. This behavioral plasticity highlights the role of appetite in fish larvae. In this study, we demonstrated that the mRNA expression of key neuropeptides in the melanocortin system responds to a meal in Atlantic salmon fry, but not in alevins. This may indicate that hunger and thus feeding behavior are not fully stimulated, and that ingestion of prey provides no or a weak signal for satiety in alevins. The fact that these neuropeptides show periprandial changes in expression in fry suggests that the feedback loops and neuronal control used to stimulate feeding behavior, ingestion, and digestion of exogenous nutrients are functional once the yolk is consumed.

The regulation of feeding behavior includes responses from peripheral tissues and central regulation in the brain. Compared to marine fish larvae, Atlantic salmon alevins have a differentiated gastric digestive system and are already able to digest formulated feed pellets from the onset of exogenous feeding, which simplifies the transition between endogenous and exogenous nutrient sources (Sahlmann et al., 2015). This is supported by the upregulation of ghrelin [mainly expressed in the stomach (Murashita et al., 2009b)], trypsin (secreted from the pancreas), cholecystokinin, leptin, and peptide yy around the first-feeding period to support the demands for developing salmon (Moen et al., 2010; Sahlmann et al., 2015). The peptides derived from the gut interact with the hypothalamic receptors on neurons expressing key neuropeptides of the melanocortin system. As a result, the salmon have a digestive system ready to digest food particles at the onset of first-feeding period, but before *npya1, pomca1*, and *a2* display periprandial responses in the brain. In general, the gene expression levels of the central melanocortin system in the brain reflect the metabolic state of an organism (Cone, 2005). However, studies have shown that the young brains of vertebrates are believed to be relatively insensitive to metabolic cues or that hypothalamic neurons fail to relay signals to other brain regions [reviewed by Coupe and Bouret (2013)].

Our results indicate that the first-feeding period is key in the development of rhythmic gene expression. However, the expression of key neuropeptides of the melanocortin system in first-feeding salmon was less in accordance with what is observed in older stages (Kalananthan et al., 2021, 2023; Tolås et al., 2021). This indicates an underdeveloped regulatory system different from that of older stages (Kalananthan et al., 2023), that requires time to became fully functional, as suggested for Senegalese sole (*Solea senegalensis*) (Bonacic et al., 2016; Navarro-Guillén et al., 2018) and Atlantic halibut (Gomes et al., 2015).

## 5. Conclusion

Our results indicate that the neuropeptides involved in the melanocortin system become actively involved in controlling feeding in Atlantic salmon when fry becomes fully dependent on exogenous feeding for energy supply. The relative expression of *npya1, pomca1*, and *pomca2* displayed a periprandial response at 991 dd when the yolk had been completely consumed, indicating that the melanocortin system plays a role in the regulation of appetite in Atlantic salmon fry and onward. Interestingly, light conditions during the development and endogenous feeding period significantly affect the periprandial mRNA levels of key appetite-controlling neuropeptides in the brain during the first-feeding window, but only for larvae reared under the LD_LD_ regime from fertilization which resembles light conditions in nature. Atlantic salmon kept under constant light conditions (DD_LD_ or LL_LD_) during development and endogenous feeding period did not display the periprandial response of any of the key neuropeptides investigated. Thus, even though growth was not affected, we postulate that fish kept under constant conditions lack a zeitgeber, in contrast to fish kept under a light-dark periodicity from fertilization. This emphasizes that the first feeding period is a key developmental stage for the development of a rhythmic expression of several genes related to appetite control.

## Data availability statement

The original contributions presented in this study are included in the article/Supplementary material, further inquiries can be directed to the corresponding author.

## Ethics statement

Ethical review and approval was not required for the animal study because the fish did not undergo handling except euthanasia. Thus, no special approval was required according to Norwegian National legislation via the Norwegian Animal Welfare Act (LOV-2015-06-09-16-65) and Regulations on the Use of Animals in Experiments (FOR-2017-04-05-451), given by the EU (Directive 2010/63/EU) for animal experiments.

## Author contributions

ME, JH, IR, AG, and SN planned and prepared the study. ME, JH, SN, and AG conducted the experiment. SN and AG performed the sampling, lab work, and statistical analyses. All authors contributed to the interpretation of the data, writing of the manuscript, read, and approved the final version.

## Footnotes

1
https://imagej.net/

2
http://www.rstudio.com/
