# Supplementary material for: Light conditions during Atlantic salmon embryogenesis affect key neuropeptides in the melanocortin system during transition from endogenous to exogenous feeding
Source: Front Behav Neurosci. 2023 Apr 21;17:1162494. doi: 10.3389/fnbeh.2023.1162494 (PMC10160384; doi:10.3389/fnbeh.2023.1162494)
Supplement: Supplementary file 1 [file Data_Sheet_1.DOCX]

***Supplemental data***

**Light conditions during Atlantic salmon embryogenesis affect key neuropeptides in the melanocortin system during transition from endogenous to exogenous feeding**

**Sissel Norland^1*^, Ana S. Gomes^1^, Ivar Rønnestad^1^, Jon Vidar Helvik^1^, Mariann Eilertsen^1^**

^1^ Department of Biological Sciences, University of Bergen, Bergen, Norway

# **Supplemental Table 1**

The sequence of specific primers used for reverse transcriptase quantitative PCR (RT-qPCR) analyses. Sequence accession number (Ensemble and GenBank databases), primer sequences, qPCR efficiency (%), and R^2^ are indicated for each primer pair.

| **Transcript** | **Acc. Number** | **Primer sequence (5’-3’)** | **Efficiency (%)** | **R^2^** |
| --- | --- | --- | --- | --- |
| *npya1* | NM_001146681 | F: GAACGCACAGCAGCAGAAAG  R: AGGATGCATATTGACTTGAAGGTT | 100 | 0.9994 |
| *npya2* | XM_014178359 | F: CAGTCCAGGTATGATGAACCGT  R: GGCACAGGAGTAACCTCTGG | 89 | 0.9999 |
| *agrp1* | NM_001146677 | F: ATGGTCATCTCAGTATTCCCAT  R: AGAGAGCCTTTACCGATATCTG | 99 | 0.9999 |
| *cart2a* | ENSSSAG00000015472 | F: GGCAAAACTGCAGGGATTGG  R: ACATAGGATGGACAGCAGCG | 95 | 0.9991 |
| *cart2b* | NM_001146680  XM_014183838 | F: TGAGAGACTTCTACCCCAAAGA  R: CGTAGGGACTTGGCCGAATT | 101 | 0.9973 |
| *cart4* | XM_014141614 | F: CGTTCCGTCGTTGGAAACAC  R: CCACGTTGGAATTGCACAGA | 97 | 0.9986 |
| *pomca1* | NM_001198575 | F: ATACTTTTGAAACAGCGTGACGA  R: CAACGAGGATTCTCCCAGCA | 101 | 0.9995 |
| *pomca2* | NM_001198576 | F: TTTGGCGACAGGCGAAGATG  R: TCCCAGCACTGACCTTTCAC | 103 | 0.9967 |
| *rps20* | NM_001140843.1 / XM_014180600.1 | F: GCAGACCTTATCCGTGGAGCTA  R: TGGTGATGCGCAGAGTCTTG | 101 | 0.9993 |

# **Supplemental Table 2**

Contrasts between light conditions, feeding time, and age derived from post-hoc test applied to generalized linear mixed model (GLMM) with gamma distribution (log-link function) for the target gene relative expression as a function of light conditions, feeding, and age.

| ***Gene*** | ***Contrast*** | ***Factor*** | ***Factor*** | ***Estimate*** | ***SE*** | ***Df*** | ***t-ratio*** | ***p-value*** |
| --- | --- | --- | --- | --- | --- | --- | --- | --- |
| *npya1* | contrast | Age2 | Light_group | estimate | SE | df | t-ratio | p-value |
|  | AF_3h - AF_6h | 991dd | LD_LD_ | 0,59 | 0,19 | 232 | 3,2E+00 | 1,6E-02 |
|  | contrast | Light_group | Feeding | estimate | SE | df | t-ratio | p-value |
|  | 830dd - 991dd | DD_LD_ | AF_1.5h | -0,43 | 0,13 | 232 | -3,3E+00 | 1,1E-03 |
|  | 830dd - 991dd | LD_LD_ | AF_1.5h | -0,43 | 0,13 | 232 | -3,3E+00 | 1,1E-03 |
|  | 830dd - 991dd | LL_LD_ | AF_1.5h | -0,43 | 0,13 | 232 | -3,3E+00 | 1,1E-03 |
|  | 830dd - 991dd | DD_LD_ | AF_3h | -0,67 | 0,13 | 232 | -5,1E+00 | 7,6E-07 |
|  | 830dd - 991dd | LD_LD_ | AF_3h | -0,67 | 0,13 | 232 | -5,1E+00 | 7,6E-07 |
|  | 830dd - 991dd | LL_LD_ | AF_3h | -0,67 | 0,13 | 232 | -5,1E+00 | 7,6E-07 |
|  | 830dd - 991dd | DD_LD_ | AF_6h | -0,42 | 0,13 | 232 | -3,3E+00 | 1,2E-03 |
|  | 830dd - 991dd | LD_LD_ | AF_6h | -0,42 | 0,13 | 232 | -3,3E+00 | 1,2E-03 |
|  | 830dd - 991dd | LL_LD_ | AF_6h | -0,42 | 0,13 | 232 | -3,3E+00 | 1,2E-03 |
|  | 830dd - 991dd | DD_LD_ | BF | -0,38 | 0,13 | 232 | -2,9E+00 | 4,0E-03 |
|  | 830dd - 991dd | LD_LD_ | BF | -0,38 | 0,13 | 232 | -2,9E+00 | 4,0E-03 |
|  | 830dd - 991dd | LL_LD_ | BF | -0,38 | 0,13 | 232 | -2,9E+00 | 4,0E-03 |
| *cart2a* | contrast | Light_group | Feeding | estimate | SE | df | t-ratio | p-value |
|  | 830dd - 991dd | DD_LD_ | AF_0.5h | -0,49 | 0,10 | 232 | -4,9E+00 | 1,8E-06 |
|  | 830dd - 991dd | LD_LD_ | AF_0.5h | -0,49 | 0,10 | 232 | -4,9E+00 | 1,8E-06 |
|  | 830dd - 991dd | LL_LD_ | AF_0.5h | -0,49 | 0,10 | 232 | -4,9E+00 | 1,8E-06 |
|  | 830dd - 991dd | DD_LD_ | AF_1.5h | -0,47 | 0,10 | 232 | -4,5E+00 | 1,3E-05 |
|  | 830dd - 991dd | LD_LD_ | AF_1.5h | -0,47 | 0,10 | 232 | -4,5E+00 | 1,3E-05 |
|  | 830dd - 991dd | LL_LD_ | AF_1.5h | -0,47 | 0,10 | 232 | -4,5E+00 | 1,3E-05 |
|  | 830dd - 991dd | DD_LD_ | AF_3h | -0,38 | 0,10 | 232 | -3,6E+00 | 3,5E-04 |
|  | 830dd - 991dd | LD_LD_ | AF_3h | -0,38 | 0,10 | 232 | -3,6E+00 | 3,5E-04 |
|  | 830dd - 991dd | LL_LD_ | AF_3h | -0,38 | 0,10 | 232 | -3,6E+00 | 3,5E-04 |
|  | 830dd - 991dd | DD_LD_ | AF_6h | -0,53 | 0,10 | 232 | -5,1E+00 | 6,5E-07 |
|  | 830dd - 991dd | LD_LD_ | AF_6h | -0,53 | 0,10 | 232 | -5,1E+00 | 6,5E-07 |
|  | 830dd - 991dd | LL_LD_ | AF_6h | -0,53 | 0,10 | 232 | -5,1E+00 | 6,5E-07 |
|  | 830dd - 991dd | DD_LD_ | BF | -0,33 | 0,10 | 232 | -3,2E+00 | 1,7E-03 |
|  | 830dd - 991dd | LD_LD_ | BF | -0,33 | 0,10 | 232 | -3,2E+00 | 1,7E-03 |
|  | 830dd - 991dd | LL_LD_ | BF | -0,33 | 0,10 | 232 | -3,2E+00 | 1,7E-03 |
| *cart2b* | contrast | Light_group | Feeding | estimate | SE | df | t-ratio | p-value |
|  | 830dd - 991dd | DD_LD_ | AF_0.5h | -0,40 | 0,10 | 232 | -3,93 | 1,1E-04 |
|  | 830dd - 991dd | LD_LD_ | AF_0.5h | -0,40 | 0,10 | 232 | -3,93 | 1,1E-04 |
|  | 830dd - 991dd | LL_LD_ | AF_0.5h | -0,40 | 0,10 | 232 | -3,93 | 1,1E-04 |
|  | 830dd - 991dd | DD_LD_ | AF_1.5h | -0,31 | 0,11 | 232 | -2,96 | 3,4E-03 |
|  | 830dd - 991dd | LD_LD_ | AF_1.5h | -0,31 | 0,11 | 232 | -2,96 | 3,4E-03 |
|  | 830dd - 991dd | LL_LD_ | AF_1.5h | -0,31 | 0,11 | 232 | -2,96 | 3,4E-03 |
|  | 830dd - 991dd | DD_LD_ | AF_3h | -0,21 | 0,10 | 232 | -1,98 | 4,9E-02 |
|  | 830dd - 991dd | LD_LD_ | AF_3h | -0,21 | 0,10 | 232 | -1,98 | 4,9E-02 |
|  | 830dd - 991dd | LL_LD_ | AF_3h | -0,21 | 0,10 | 232 | -1,98 | 4,9E-02 |
|  | 830dd - 991dd | DD_LD_ | AF_6h | -0,32 | 0,10 | 232 | -3,13 | 2,0E-03 |
|  | 830dd - 991dd | LD_LD_ | AF_6h | -0,32 | 0,10 | 232 | -3,13 | 2,0E-03 |
|  | 830dd - 991dd | LL_LD_ | AF_6h | -0,32 | 0,10 | 232 | -3,13 | 2,0E-03 |
|  | 830dd - 991dd | DD_LD_ | BF | -0,36 | 0,10 | 232 | -3,41 | 7,6E-04 |
|  | 830dd - 991dd | LD_LD_ | BF | -0,36 | 0,10 | 232 | -3,41 | 7,6E-04 |
|  | 830dd - 991dd | LL_LD_ | BF | -0,36 | 0,10 | 232 | -3,41 | 7,6E-04 |
| *cart4* | contrast | Light_group | Feeding | estimate | SE | df | t-ratio | p-value |
|  | 830dd - 991dd | DD_LD_ | AF_0.5h | 0,21 | 0,10 | 232 | 2,12 | 3,5E-02 |
|  | 830dd - 991dd | LD_LD_ | AF_0.5h | 0,21 | 0,10 | 232 | 2,12 | 3,5E-02 |
|  | 830dd - 991dd | LL_LD_ | AF_0.5h | 0,21 | 0,10 | 232 | 2,12 | 3,5E-02 |
| *pomca1* | contrast | Age2 | Light_group | estimate | SE | df | t-ratio | p-value |
|  | AF_1.5h - BF | 991dd | LD_LD_ | 0,75 | 0,24 | 232 | 3,19 | 1,4E-02 |
|  | AF_3h - BF | 991dd | LD_LD_ | 0,81 | 0,24 | 232 | 3,39 | 7,3E-03 |
|  | AF_6h - BF | 991dd | LD_LD_ | 0,69 | 0,24 | 232 | 2,89 | 3,4E-02 |
|  | contrast | Light_group | Feeding | estimate | SE | df | t-ratio | p-value |
|  | 830dd - 991dd | DD_LD_ | AF_1.5h | -0,47 | 0,16 | 232 | -2,93 | 3,7E-03 |
|  | 830dd - 991dd | LD_LD_ | AF_1.5h | -0,47 | 0,16 | 232 | -2,93 | 3,7E-03 |
|  | 830dd - 991dd | LL_LD_ | AF_1.5h | -0,47 | 0,16 | 232 | -2,93 | 3,7E-03 |
|  | 830dd - 991dd | DD_LD_ | AF_3h | -0,36 | 0,16 | 232 | -2,25 | 2,5E-02 |
|  | 830dd - 991dd | LD_LD_ | AF_3h | -0,36 | 0,16 | 232 | -2,25 | 2,5E-02 |
|  | 830dd - 991dd | LL_LD_ | AF_3h | -0,36 | 0,16 | 232 | -2,25 | 2,5E-02 |
|  | 830dd - 991dd | DD_LD_ | AF_6h | -0,64 | 0,16 | 232 | -3,99 | 9,0E-05 |
|  | 830dd - 991dd | LD_LD_ | AF_6h | -0,64 | 0,16 | 232 | -3,99 | 9,0E-05 |
|  | 830dd - 991dd | LL_LD_ | AF_6h | -0,64 | 0,16 | 232 | -3,99 | 9,0E+00 |
| *pomca2* | contrast | Feeding | Age2 | estimate | SE | df | t-ratio | p-value |
|  | LD_LD_ - LL_LD_ | AF_0.5h | 830dd | -0,49 | 0,18 | 232 | -2,75 | 1,77E-02 |
|  | DD_LD_ - LD_LD_ | BF | 830dd | 0,60 | 0,19 | 232 | 3,14 | 5,40E-03 |
|  | LD_LD_ - LL_LD_ | BF | 830dd | -0,76 | 0,19 | 232 | -3,94 | 3,16E-04 |
|  | LD_LD_ - LL_LD_ | AF_0.5h | 991dd | -0,49 | 0,18 | 232 | -2,75 | 1,77E-02 |
|  | DD_LD_ - LD_LD_ | BF | 991dd | 0,60 | 0,19 | 232 | 3,14 | 5,40E-03 |
|  | LD_LD_ - LL_LD_ | BF | 991dd | -0,76 | 0,19 | 232 | -3,94 | 3,16E-04 |
|  | contrast | Age2 | Light_group | estimate | SE | df | t-ratio | p-value |
|  | AF_0.5h - BF | 991dd | LD_LD_ | 0,62 | 0,22 | 232 | 2,78 | 4,61E-02 |
|  | AF_1.5h - BF | 991dd | LD_LD_ | 0,87 | 0,23 | 232 | 3,80 | 1,72E-03 |
|  | AF_3h - BF | 991dd | LD_LD_ | 0,88 | 0,23 | 232 | 3,80 | 1,70E-03 |
|  | AF_6h - BF | 991dd | LD_LD_ | 0,70 | 0,23 | 232 | 3,07 | 2,02E-02 |
|  | contrast | Light_group | Feeding | estimate | SE | df | t-ratio | p-value |
|  | 830dd - 991dd | DD_LD_ | AF_1.5h | -0,46 | 0,15 | 232 | -3,05 | 2,58E-03 |
|  | 830dd - 991dd | LD_LD_ | AF_1.5h | -0,46 | 0,15 | 232 | -3,05 | 2,58E-03 |
|  | 830dd - 991dd | LL_LD_ | AF_1.5h | -0,46 | 0,15 | 232 | -3,05 | 2,58E-03 |
|  | 830dd - 991dd | DD_LD_ | AF_3h | -0,38 | 0,15 | 232 | -2,49 | 1,37E-02 |
|  | 830dd - 991dd | LD_LD_ | AF_3h | -0,38 | 0,15 | 232 | -2,49 | 1,37E-02 |
|  | 830dd - 991dd | LL_LD_ | AF_3h | -0,38 | 0,15 | 232 | -2,49 | 1,37E-02 |
|  | 830dd - 991dd | DD_LD_ | AF_6h | -0,69 | 0,15 | 232 | -4,51 | 1,01E-05 |
|  | 830dd - 991dd | LD_LD_ | AF_6h | -0,69 | 0,15 | 232 | -4,51 | 1,01E-05 |
|  | 830dd - 991dd | LL_LD_ | AF_6h | -0,69 | 0,15 | 232 | -4,51 | 1,01E-05 |

# **Supplemental Table 3**

The number of Atlantic salmon per age, light condition, and sampling time point before (BF) and after (AF) the first meal of the day.

| **Age** | **Light** | **BF 1h** | **AF 0.5h** | **AF 1.5h** | **AF 3h** | **AF 6h** | **Total** |
| --- | --- | --- | --- | --- | --- | --- | --- |
| 830dd | DD_LD_ | 9 | 8 | 7 | 8 | 9 | 41 |
|  | LD_LD_ | 8 | 9 | 7 | 8 | 9 | 41 |
|  | LL_LD_ | 8 | 9 | 9 | 8 | 8 | 42 |
|  | Total | 25 | 26 | 23 | 24 | 26 | 124 |
| 991dd | DD_LD_ | 9 | 9 | 9 | 9 | 8 | 44 |
|  | LD_LD_ | 7 | 9 | 9 | 8 | 9 | 42 |
|  | LL_LD_ | 9 | 9 | 9 | 8 | 9 | 44 |
|  | Total | 25 | 27 | 27 | 25 | 26 | 130 |


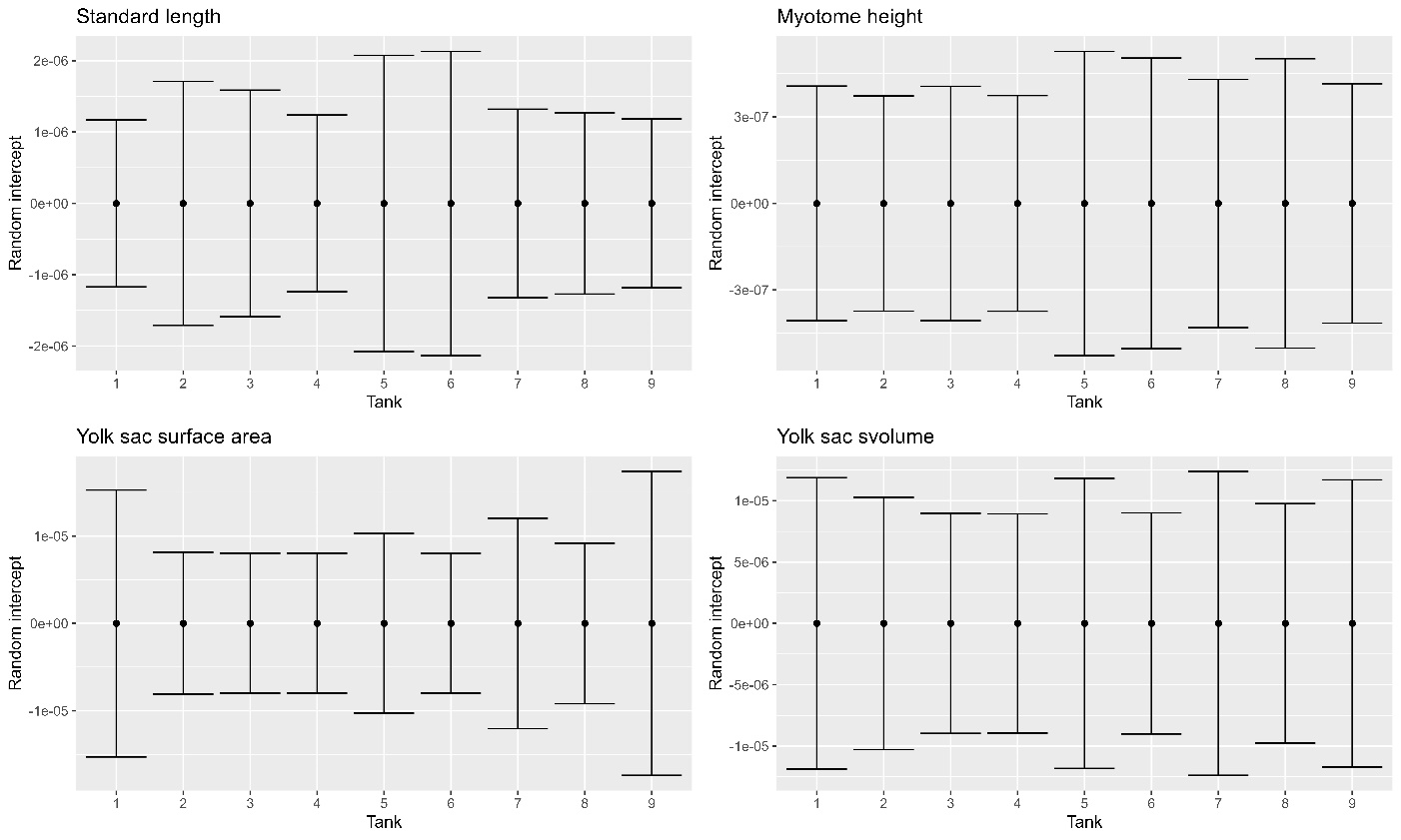


**Supplemental figure 1**. Random intercept for the modeled biometric data with tank effect.


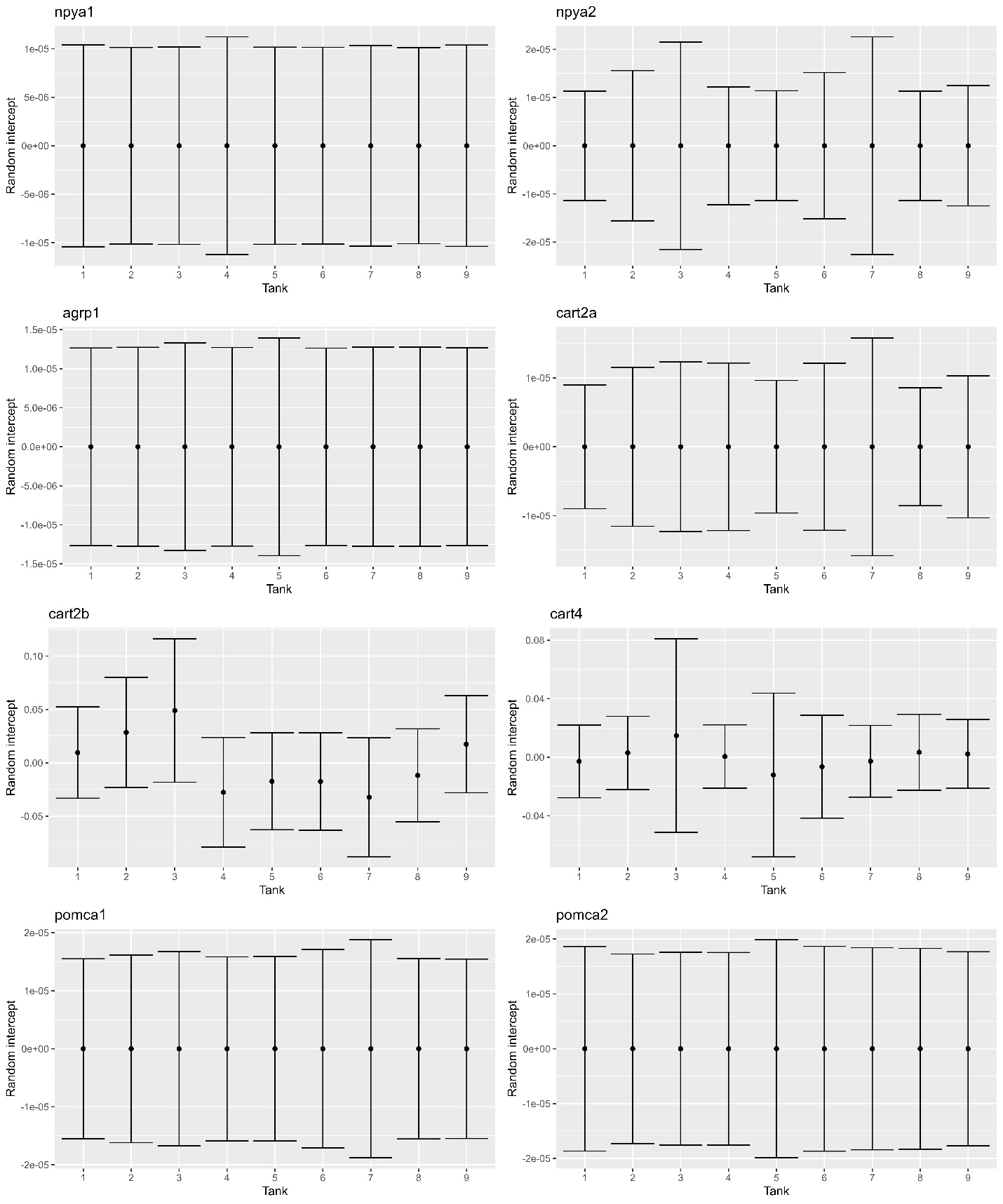


**Supplemental Figure 2**. Random intercept for the modeled mRNA expression with tank effect.


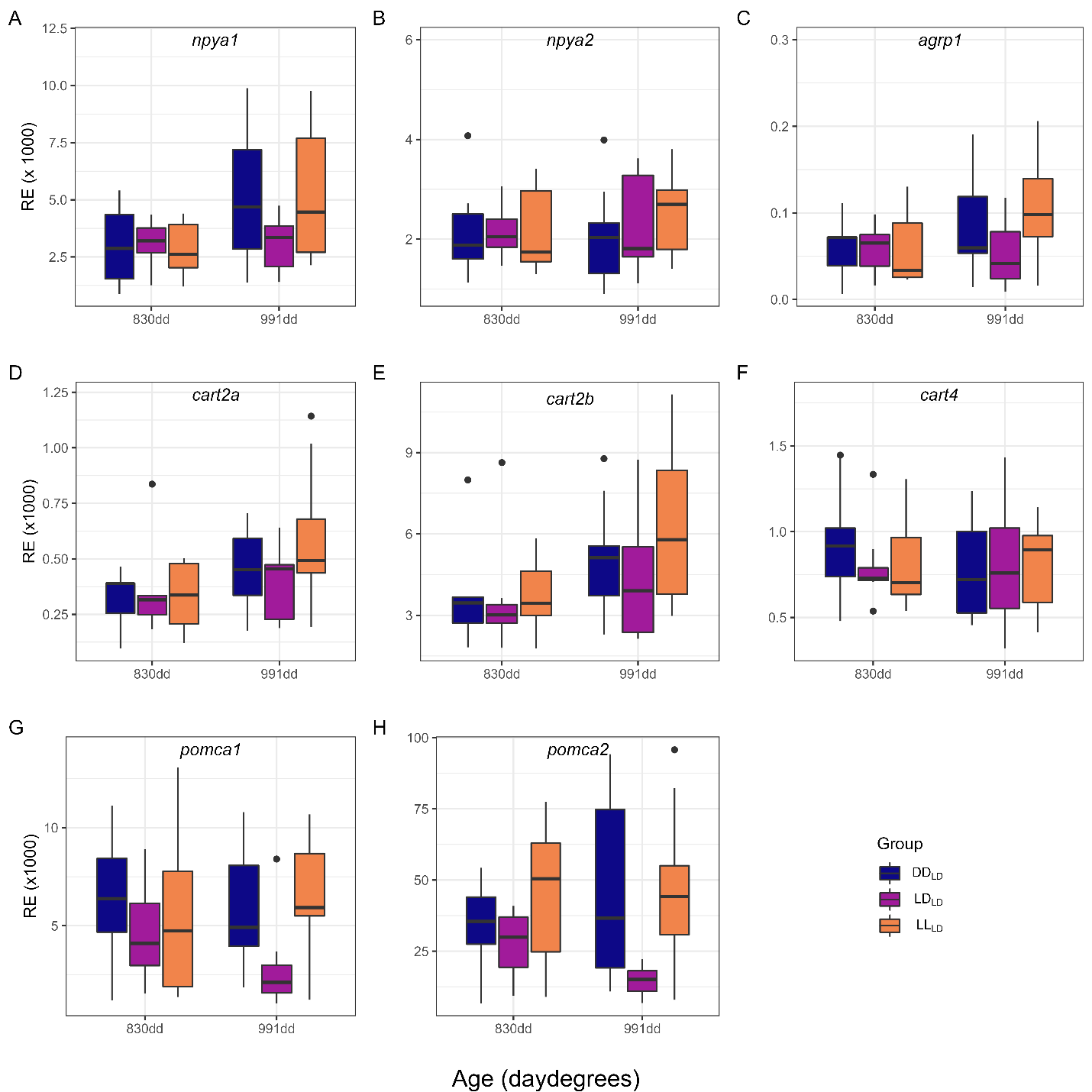


**Supplemental Figure 3**. The relative expression of target genes in first feeding Atlantic salmon at 830- and 991-day degrees (dd) 1 hour before the first meal of the day. Number per light condition and age group n = 9, except for the groups in Supplemental Table 2. RE: Relative expression (× 1000).
